# Vaccination of recurrent glioma patients with tumour lysate-pulsed dendritic cells elicits immune responses: results of a clinical phase I/II trial

**DOI:** 10.1038/sj.bjc.6601268

**Published:** 2003-09-30

**Authors:** R Yamanaka, T Abe, N Yajima, N Tsuchiya, J Homma, T Kobayashi, M Narita, M Takahashi, R Tanaka

**Affiliations:** 1 Department of Neurosurgery, Brain Research Institute, Niigata University, Asahimachi-dori 1-757, Niigata City, 951-8585, Japan; 2 Department of First Internal Medicine, Niigata University School of Medicine, Niigata University, Niigata, Japan

**Keywords:** glioma, dendritic cell, immunotherapy, ELISPOT assay

## Abstract

In this Phase I/II trial, the patient's peripheral blood dendritic cells were pulsed with an autologous tumour lysate of the glioma. Seven patients with glioblastoma and three patients with anaplastic glioma, ranging in age from 20 to 69 years, participated in this study. The mean numbers of vaccinations of tumour lysate-pulsed dendritic cells were 3.7 times intradermally close to a cervical lymph node, and 3.2 times intratumorally via an Ommaya reservoir. The percentage of CD56-positive cells in the peripheral blood lymphocytes increased after immunisation. There were two minor responses and four no-change cases evaluated by radiological findings. Dendritic cell vaccination elicited T-cell-mediated antitumour activity, as evaluated by the ELISPOT assay after vaccination in two of five tested patients. Three patients showed delayed-type hypersensitivity reactivity to the autologous tumour lysate, two of these had a minor clinical response, and two had an increased ELISPOT result. Intratumoral CD4+ and CD8+ T-cell infiltration was detected in two patients who underwent reoperation after vaccination. This study demonstrated the safety and antitumour effects of autologous tumour lysate-pulsed dendritic cell therapy for patients with malignant glioma.

Despite advances in radiation and chemotherapy along with surgical resectioning, the prognosis for patients with malignant glioma is poor. Among the new treatments currently being investigated for malignant glioma, immunotherapy is theoretically very attractive, since it offers the potential for high tumour-specific cytotoxicity (Dranoff *et al*, 1993; Sampson *et al*, 1996; Yu *et al*, 2001). Previous immunotherapeutic treatments for brain tumours have focused on passive, adoptive, and nonspecific strategies, and have failed to yield clear benefits. The central nervous system (CNS) has been considered to be immunologically privileged based on the fact that tissue or tumour allografts survive better in the brain than in extracerebral locations (Medawar, 1948). Glioma cells are poor antigen-presenting cells (APCs) for the immune system because of the downregulation in glioma cells of costimulatory molecules required to activate the immune system (Satoh *et al*, 1995), and the secretion by glioma cells of immunosuppressive cytokines such as transforming growth factor-*β* (TGF-*β*) (Constam *et al*, 1992), vascular endothelial growth factor (VEGF) (Gabrilovich *et al*, 1999), and interleukin (IL)-10 (Chen *et al*, 1994). However, subcutaneous vaccination with genetically modified cytokine-secreting tumour cells has been demonstrated to be efficacious against intracranial tumours in a murine model, which supports the notion that an effective immune response can be generated against intracerebral tumours (Dranoff *et al*, 1993; Sampson *et al*, 1996). There are an increasing number of reports (Liau *et al*, 1999, 2000; Heimberger *et al*, 2000; Akasaki *et al*, 2001; Aoki *et al*, 2001; Kikuchi *et al*, 2001; Ni *et al*, 2001; Yamanaka *et al*, 2001, 2002; Yu *et al*, 2001; Insug *et al*, 2002; Witham *et al*, 2002) demonstrating that systemic immunotherapy using dendritic cells (DCs) is capable of inducing an antitumour response within the immunologically privileged brain, confirming that the CNS may not be an absolute barrier to DC-based immunotherapy. Dendritic cells are rare, haematopoietically derived leucocytes that form a cellular network involved in immune surveillance, antigen capture, and antigen presentation (Steinman *et al*, 1997). With the availability of techniques for the isolation and bulk propagation of DCs *in vitro*, great efforts have been made to use DCs in various immunisation strategies (Fong and Engleman, 2000). Many strategies for delivering antigens into DCs have been established in murine models and are now undergoing evaluation in clinical trials. These include the use of synthetic peptides where the tumour antigen is known (Mayordomo *et al*, 1995), stripped peptides derived from class I molecules from tumours (Tjoa *et al*, 1998), tumour RNA (Boczkowski *et al*, 1996), and tumour lysates (Heimberger *et al*, 2000; Aoki *et al*, 2001; Ni *et al*, 2001), and fusing DCs and tumour cells as a vaccine (Gong *et al*, 1997, 2000). The use of synthetic peptide approaches requires identification of tumour-specific antigens for individual tumours and demonstration of their recognition by cytotoxic T-lymphocytes (CTLs), a process that is difficult. To date, therefore, there has been limited identification of the antigenic peptides and CTL epitopes presented by human gliomas (Yamanaka *et al*, 2003). The advantages of vaccinating with total tumour-derived material, such as tumour cell lysates or tumour-derived mRNA, are that the identities of tumour antigens need not be known and that the use of multiple tumour antigens reduces the risk of antigen-negative escape mutants. The administration of DCs therapy has mainly been through the intradermal or intranodal route. Intratumorally injected DCs expressing IL-12 migrated to the draining lymph node and induced therapeutic antitumour immunity (Nishioka *et al*, 1999). We have investigated both intradermal and intratumoral injection of DCs in this study. Here, we describe the vaccination of 10 malignant glioma cases with DCs pulsed by an autologous tumour lysate. The safety and immunological responses of this study are discussed. Systemic cytotoxicity and intratumoral T-cell infiltration were elicited in some of the patients.

## MATERIALS AND METHODS

### Patient population

There were 10 patients enrolled in this Phase I/II study: six women and four men with an age range from 20 to 69 years (average, 46. 1 years; Table 1

**Table 1:** Patient characteristics

**Case**	**Age/sex**	**Pathological diagnosis**	**KPS**
1	46M	Glioblastoma	30
2	40F	Glioma	40
3	53F	Glioblastoma	80
4	47F	Anaplastic mixed glioma	40
5	60M	Glioblastoma	30
6	65F	Glioblastoma	80
7	69M	Glioblastoma	40
8	20F	Glioblastoma	80
9	34M	Anaplastic oligoastrocytoma	60
10	27F	Glioblastoma	70

). Patients had histologically proven glioblastoma, anaplastic astrocytoma, or other malignant gliomas according to the World Health Organization (WHO) criteria. After surgical resection of their tumour, patients had a course of external beam radiation therapy (standard dose, 40 Gy to the tumour with 3-cm margins, 20 Gy boost to the whole brain). Patients were monitored for recurrence of their tumour after the initial and maintenance therapy by magnetic resonance imaging (MRI) or computed tomography (CT), and had no chemotherapy or radiotherapy during the previous 4 weeks. The patients started receiving DC immunotherapy when the recurrence was detected. Treatments were carried out at the Department of Neurosurgery, Niigata University Hospital. Five patients (Cases 2, 4, 5, 7, and 9 in Table 1) had a maintenance dose (predonine, 30 mg day^−1^) of glucocorticoid therapy during the immunotherapy. The median Karnofsky performance scale was 54, ranging from 30 to 80. Exclusion criteria included pulmonary, cardiac, or other systemic disease, an acute infection, and a history of an autoimmune disease.

### Generation of dendritic cells from peripheral blood

A concentrated 100 ml leucocyte fraction was generated through a 1-h restricted peripheral blood leukapheresis processing 3–4 l of blood with each collection. Peripheral blood mononuclear cells (PBMCs) were then purified using Ficoll-Hypaque (Sigma, Tokyo) density gradient centrifugation. Peripheral blood mononuclear cells were resuspended in RPMI-1640 (Invitrogen, Tokyo) with 1% autologous heat-inactivated serum, plated at a concentration of 5 × 10^6^ cells ml^−1^ and allowed to adhere to 10 cm^2^ dishes. Nonadherent cells were removed after 4 h at 37°C in a humidified 5% CO_2_/95% air incubator, and the adherent cells were cultured at 37°C for 7 days in RPMI-1640 medium supplemented with 1% heat-inactivated autologous serum, in the presence of 1000 U ml^−1^ recombinant human granulocyte – macrophage colony-stimulating factor (GM-CSF; Immunex Corp., Seattle, WA, USA), 500 U ml^−1^ recombinant human IL-4 (R&D Systems, Inc., Minneapolis, MN, USA), and 1% penicillin/streptomycin (Invitrogen, Tokyo). After 7 days of culture, semiadherent and nonadherent cells were harvested by pipetting and used as dendritic cells for pulsing with the tumour lysate as described below. The DCs were stained with anti-human CD3, CD4, CD8, CD14, CD16, CD19, CD40, CD86, CD80, CD83, CD56, MHC I, and MHC II monoclonal antibodies (Pharmingen, San Diego, CA, USA) for 30 min at 4°C. Species- and isotype-matched monoclonal antibodies were used as controls.

### Preparation of tumour lysate

Tumour tissue was removed from the vivid glioma portion seen in the microsurgical scope at the operation, and immediately placed in phosphate-buffered saline (PBS). Adjacent nonglioma tissue was removed using a scalpel, and tumour cells were dispersed to create a single-cell suspension. Aliquots were taken for cell counting and viability staining by trypan blue exclusion. Cells were lysed by three to five freeze cycles in liquid nitrogen and thaw cycles at room temperature. Lysis was monitored by light microscopy. Large particles were removed by centrifugation (15 min, 400 **g**), and the supernatants were passed through a 0.45 *μ*m filter. The protein contents were determined and aliquots were stored at −80°C until use.

### DC pulsing

Following 7 days of culture, DCs were further cultured overnight with 50 *μ*g ml^−1^ keyhole limpet haemocyanin (KLH) (Calbiochem, Bad Soden, Germany) and the tumour lysate. Approximately 1 × 10^7^ DCs were cultured with 725 *μ*g of autologous tumour lysate. The cells were washed three times with PBS and resuspended in RPMI-1640 as described below.

### Design of the phase I/II trial of DC therapy (Figure 1)

**Figure 1:**
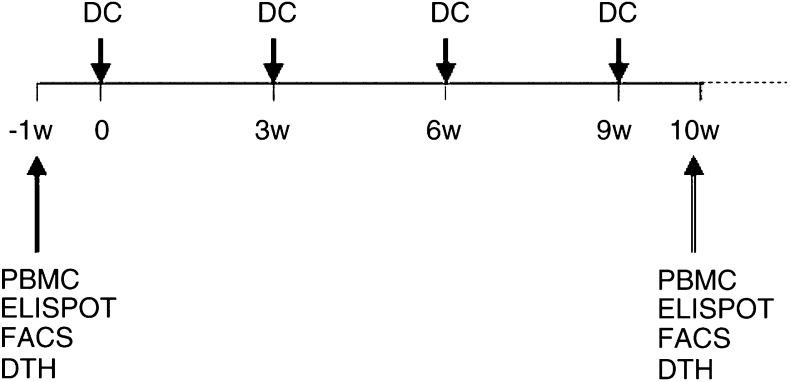
Flow chart of the phase I/II trial of DC therapy.

The study protocol was approved by the Ethical Committee of Niigata University. All patients provided informed consent before treatment. Patients received the DCs pulsed with the autologous tumour lysate every 3 weeks for a minimum of one, and a maximum of 10, immunisations (Table 2

**Table 2:** Results of dendritic-cell (DC) immunotherapy

**Case**	**No. of vaccination**	**Total amount of DCs (× 10^6^)**	**Radiological findings**	**Adverse effects**	**Overall survival (weeks)**	**Outcome**
1	2(id)	10(id)	NC	No	73	Dead
2	2(id), 2(it)	17(id), 17(it)	NC	No	216	Dead
3	4(id), 4(it)	42.4(id),52.4(it)	MR	No	100	Dead
4	1(id), 1(it)	12.6(id),12.6(it)	NC	No	188	Dead
5	2(id), 2(it)	64(id), 64(it)	PD	No	63	Dead
6	10(id), 7(it)	137.2(id),106.6(it)	MR	Grade 1	308+	SD
7	3(id)	46(id)	PD	No	52	Dead
8	6(id)	37.5(id)	NC	No	60	Dead
9	4(id)	28.2(id)	PD	No	98	Dead
10	3(id)	20.6(id)	PD	No	564+	PD

SD=stable disease; PR=partial response; MR=minor response; NC=no change; PD=progressive disease; id=intradermal vaccination; it=intratumoral vaccination.

). Eligible patients received four vaccinations at 3 weekly intervals. The immunisation was subsequently continued for up to 10 vaccinations, depending on the clinical response. Dendritic cells ranging from 10 × 10^6^ to 32 × 10^6^ cells were injected per vaccination. Dendritic cells were injected intradermally close to a cervical lymph node and/or intratumorally via an Ommaya reservoir. Those patients who had a settled Ommaya reservoir at an appropriate tumour cavity received an intratumoral DC injection. Patients were monitored for immediate and delayed toxicities. All toxicity was graded using the National Cancer Institute Common Toxicity Criteria. The overall survival time in weeks was estimated from the onset of disease to death or the present time. The response to the treatment was evaluated by clinical observations and radiological findings. Magnetic resonance imaging or CT scanning was performed to evaluate the intracranial lesions after the vaccinations every month. Tumour size was estimated as the volume of the region of abnormal enhancement observed on MRI or CT via direct measurement. Responses were classified into the following categories based on Macdonald's criteria (Macdonald *et al*, 1990): (a) complete response (CR), defined as disappearance of the entire tumour for a period of at least 4 weeks; (b) partial response (PR), defined as a reduction of 50% or more in the tumour size for at least 4 weeks; (c) minor response (MR), defined as a 25–50% decrease of lesions lasting at least 4 weeks or a more than 50% decrease of lesions lasting less than 4 weeks; (d) no change (NC), defined as either a decrease of less than 25% or an increase of less than 25% in tumour size for at least 4 weeks; (e) progressive disease (PD), defined as an increase of 25% or more in tumour size. The end points of this study were evaluations of the toxicity, immunological response, and clinical response after DC therapy.

### Delayed-type hypersensitivity (DTH) reaction

To test the cell-mediated cytotoxicity response, 0.05 *μ*g purified tuberculin and 10 *μ*g autologous tumour lysate were administered intradermally into the forearm before and after treatment. A positive DTH skin-test reaction was defined as >2 mm diameter induration after 48 h.

### IFN-*γ* ELISPOT assay

Wells of MultiScreen-HA plates (Millipore, Bedford, MA, USA) were coated with 100 *μ*l of a mouse anti-human IFN-*γ* antibody (2 *μ*g ml^−1^, clone 1-D1K; Mabtech, Nacka, Sweden) in PBS. After incubation overnight at 4°C, unbound antibody was removed by six washes with PBS. Coated wells were blocked with 100 *μ*l RPMI-1640 supplemented with 1% bovine serum albumin (BSA; Sigma). After 2 h at 37°C, the blocking medium was discarded. Mononuclear cells (2 × 10^5^ well^−1^ were used as stimulator cells and plated in each well. Tumour lysate-pulsed DCs (1 × 10^4^ well^−1^ were admixed to each well. After a 6-h incubation at 37°C, cells were removed by six washes with PBS plus 0.05% Tween 20 (PBST), and 100 *μ*l of a biotinylated detection antibody against human IFN-*γ* (0.75 *μ*g/ml^−1^, clone 7-B6-1; Mabtech) was added per well. After 20 h at 37°C, plates were rinsed six times with PBST. Then, 100 *μ*l of a streptavidin-alkaline phosphatase complex preparation (Mabtech) was added to each well at a dilution of 1 : 1000 and incubated for 1 h at room temperature. Unbound complexes were removed by three washes with PBST and three washes with PBS. The staining reaction was initiated by adding a solution of 5-bromo-4-chloro-3-indolyl-phosphate (BCIP)/nitroblue tetrazolium (NBT) (BIO-RAD Lab., Hercules, CA, USA) dissolved according to the manufacturer's instructions. The reaction was stopped after 5 min by washing the plates under running tap water. Spots were counted using a stereomicroscope (Zeiss, Jena, Germany) at × 40 magnification. The cutoff for positive spots was defined as a spot size greater than 3 × s.d. above the mean value of the spot diameter obtained in the absence of the DCs.

### Cell surface analysis

Peripheral blood mononuclear cells were separated from the peripheral blood of patients, resuspended in PBS containing 1% BSA and 0.1% sodium azide (Sigma), and stained with anti-human CD3, CD4, CD8, CD14, CD16, CD19, CD40, CD86, CD80, CD83, CD56, MHC I, and MHC II monoclonal antibodies (Pharmingen, San Diego, CA, USA) for 30 min at 4°C. Stained cells were washed and analysed using FACScan (Becton Dickinson, San Jose, CA, USA).

### Immunohistochemistry

Serial 10 *μ*m paraffin sections of the surgical intracranial tumour specimen were stained with mouse anti-human monoclonal antibodies against CD3, CD4, CD8, CD20, and CD56 (1 : 50 dilutions; Pharmingen). Primary antibodies were detected with an FITC-conjugated goat anti-mouse IgG secondary antibody (1 : 500 dilution; Pharmingen). Sections were examined in a Zeiss Axiophot 2 fluorescence microscope (Zeiss).

### Statistical analysis

The pre- and postvaccination data were compared using Wilcoxon's test. Statistical significance was determined at the <0.05 level.

## RESULTS

### Isolation and characterisation of DCs

Mononuclear cells (0.35–9 × 10^8^; average, 4.9 × 10^8^) were isolated by Ficoll-Hypaque density gradient centrifugation and differentiated into DCs in the presence of GM-CSF and IL-4. The final yield of DCs after 7 days of culture was 0.3–4 × 10^8^ (average, 1.36 × 10^8^). Dendritic cells of the immature phenotype (CD3neg, CD14neg, CD16neg/CD56neg, CD19neg, MHC I pos, MHC II pos, CD40low, CD86low, and CD83low) were greater than 75% (data not shown). These DCs were divided into six tubes, and cryopreserved in 90% RPMI-1640 supplemented with 10% autoplasma+10% DMSO in liquid nitrogen. One cryopreserved vial was thawed and used for each vaccination. The DCs were tested for endotoxin and mycoplasma prior to administration to the patient.

### Safety of DC therapy

Intradermal and/or intratumoral vaccination with DCs was performed. The mean numbers of administrations were 3.7 times intradermally, and 3.2 times intratumorally, ranging from 1 to 10. The mean total numbers of inoculated DCs were 4.155 × 10^7^ cells for intradermal injection, and 5.052 × 10^7^ cells for intratumoral injection (Table 2). There were no serious adverse effects, clinical or radiological evidence of autoimmune reaction, in any of the patients (Table 2). There were no substantial changes in the results of routine blood tests (data not shown). Patient 6 developed a mild headache lasting a few days after vaccination. In two cases (Cases 4 and 6), mild erythema at the cervical injection site manifested after the third immunisation, suggesting that DTH had occurred.

### Delayed-type hypersensitivity reactivity

Delayed-type hypersensitivity, using purified tuberculin or the autologous tumour lysate, was performed before and after treatment. Three of five patients showed reactivity to the purified tuberculin before and after vaccination. Three of six patients showed reactivity to the autologous tumour lysate; two of these had a minor reaction on MRI or CT, and two had an increased ELISPOT result after vaccination. Two of three patients who showed reactivity to the autologous tumour lysate also exhibited reactivity to purified tuberculin.

### Clinical responses

**Figure 2:**
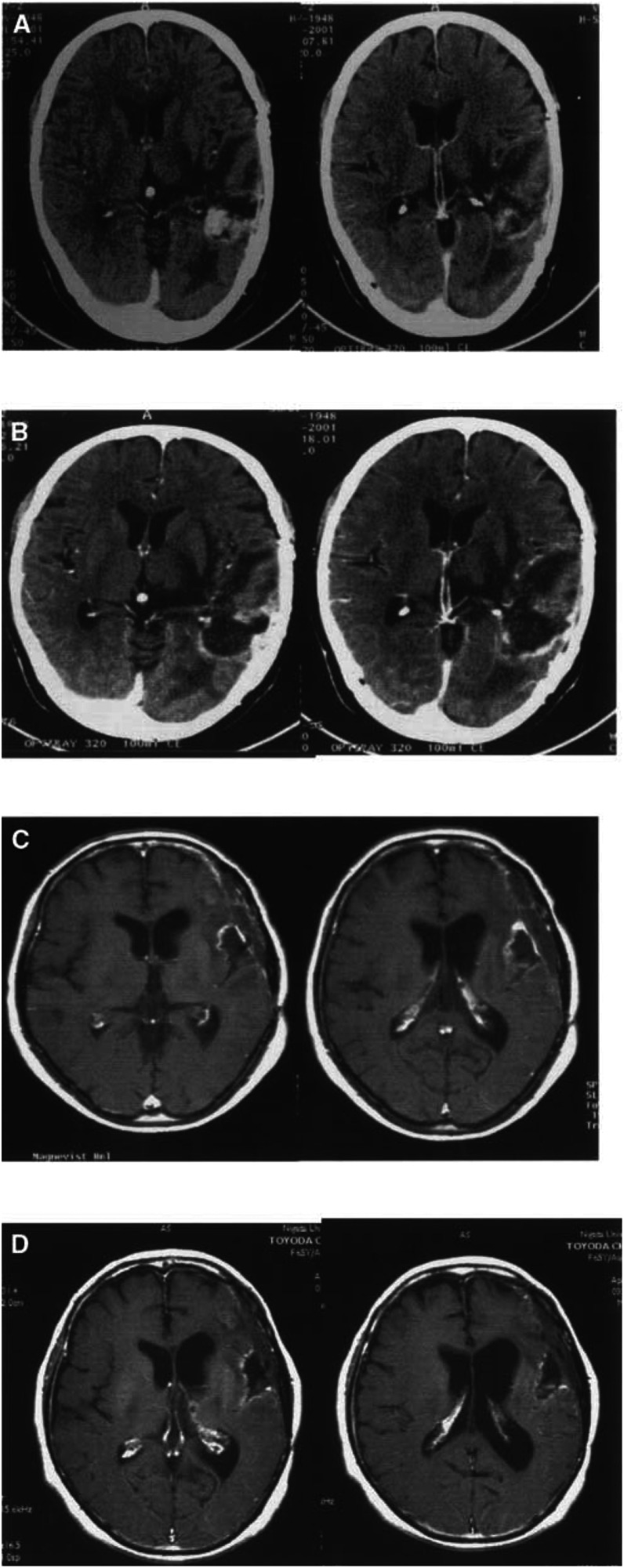
Computed tomography of Case 3 shows that, although the size of the low density area did not change, the contrast enhanced lesion was decreased. Computed tomography contrast enhanced images before (**A**) and after (**B**) vaccination. Magnetic resonance imaging of Case 6 shows that the contrast enhanced lesion was decreased after vaccination. Contrast enhanced MRI before (**C**) and after (**D**) vaccination.

The clinical response data are listed in Table 2. There were two minor responses (Cases 3 and 6). In Case 3, although the size of the low density area did not decrease, the contrast enhanced lesion was decreased (Figure 2A, B

). Case 6 had convulsions that worsened at the beginning of immunisation. The patient showed a slight improvement in the convulsions after the immunisations, and MRI showed a reduction in the size of enhanced lesions of the tumour (Figure 2C, D). There were four no-change cases on MRI.

### CD56-positive cells in PBL increased after vaccination

The surface phenotype of the peripheral blood lymphocytes was investigated using FACScan before and after immunotherapy. We analysed the expressions of CD3, CD4, CD8, CD16, CD19, and CD56 in five of 10 cases. No constant changes in the percentages of CD3 or CD4 were detected in the periods before and after therapy, whereas the percentages of CD8-, CD16-, and CD19-positive cells slightly increased in four of five cases; CD56-positive cells slightly increased in all cases analysed (Table 3

**Table 3:** Surface phenotypes of PBLs pre- and postvaccination with dendritic cells

	**CD3(%)**		**CD4(%)**		**CD8(%)**		**CD16(%)**		**CD19(%)**		**CD56(%)**	
**Case**	**Pre**	**Post**	**Pre**	**Post**	**Pre**	**Post**	**Pre**	**Post**	**Pre**	**Post**	**Pre**	**Post**
3	70.38	63.32	37.52	27.16	30.02	37.52	6.4	7.92	20.26	23.46	19.34	23.2
5	41.34	41.96	30.9	31.28	24.84	43.6	41.24	33.82	11.32	17	47.42	53.06
6	66	66.56	36.3	29.8	39.8	48.14	17.5	19.92	5.5	3.34	28.1	28.36
7	69.14	64.28	35.14	28.22	47.64	47.14	15.88	17.52	11.98	15.5	20.14	23.64
8	78.68	71.78	45.16	40.48	35.26	43.04	2.9	15.28	6.44	24.44	15.36	22.18

). There was a significant increase in CD56-positive cells in PBL after vaccination (*P*<0.05).

### Detection of tumour lysate-reactive CD8+ T cells in the blood of glioma patients

**Figure 3:**
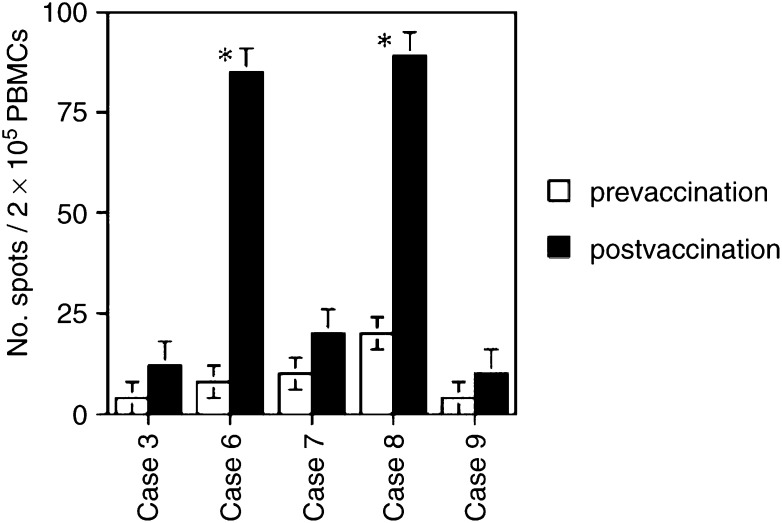
T-cell-mediated antitumour activity of PBMCs of glioma patients, as evaluated by the ELISPOT assay before and after vaccination. Data are shown as the median values±standard deviation (*n*=3). Statistical significance was assessed by Student's *t*-test. ^*^P<0.05.

By ELISPOT analysis, blood samples of five glioma patients were tested for the presence of tumour lysate-reactive CD8+ T cells in PBMC from patients’ prevaccination, and 1 week after the last vaccination. To determine the frequency of tumour lysate-reactive CD8+ T cells by the IFN-*γ* ELISPOT assay, monocytes were prepared from the blood of the five glioma patients, loaded with tumour lysate-pulsed DCs, and then cocultured. As a negative control, PBMCs were not pulsed with DCs. The results of the ELISPOT analysis are summarised in Figure 3

. T cells reactive against tumour lysate-pulsed DCs were increased in two patients after vaccination (*P*<0.05). On the other hand, only weak T-cell responses against tumour lysate-pulsed DCs were observed in three glioma patients after vaccination.

### Intracranial infiltration of T cells after vaccination

**Figure 4:**
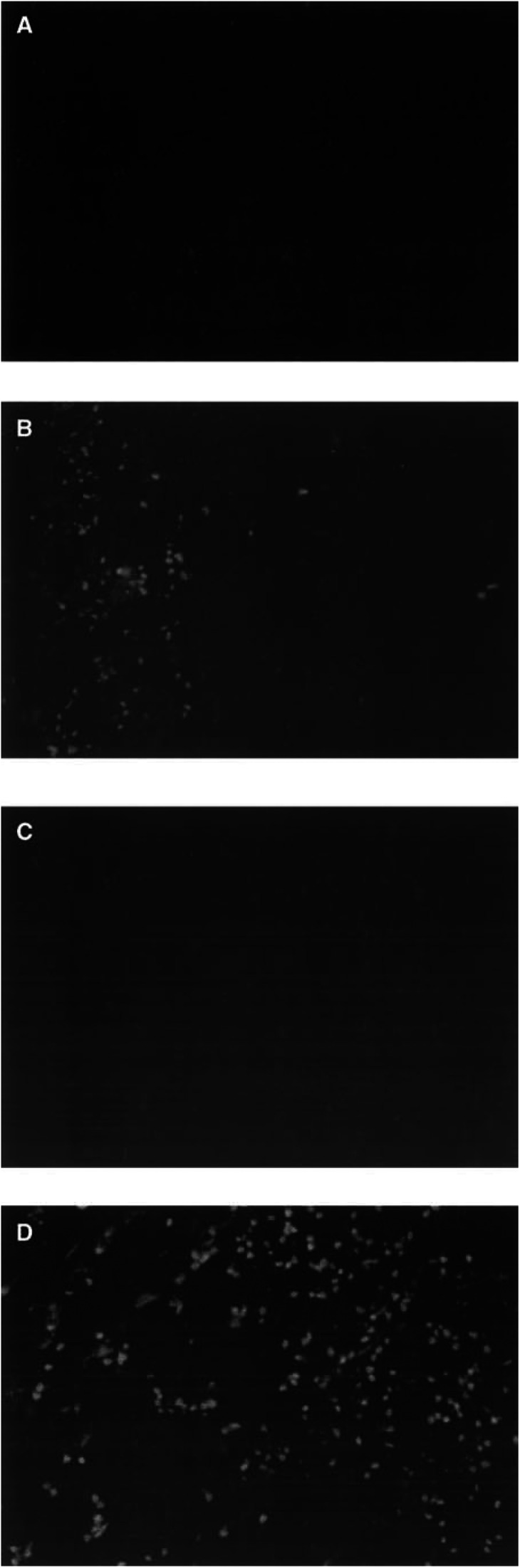
Immunohistochemical characterisation of infiltrating cells in an intracranial tumour at first surgery, before vaccination and at reoperation after vaccination. (**A**) CD4+ T cell before vaccination; (**B**) CD4+ T cell after vaccination; (**C**) CD8+ T cell before vaccination; (**D**) CD8+ T-cell after vaccination (magnification × 400).

Two patients (Cases 8 and 10) who developed tumour progression, as suggested by new areas of contrast enhancement on MRI or CT, underwent reoperation subsequent to the third intradermal vaccination. These patients developed CD4+ and CD8+ T-cell infiltration in areas of the tumour that were not apparent in the tumour specimen obtained before vaccination (Figure 4

). There were no CD20+ B cells detectable before or after vaccination (data not shown). There were no increases in the number of intratumoral lymphocytes in specimens from five nonvaccinated glioblastoma patients who underwent reoperation (data not shown). Thus, enhanced CD4+ and CD8+ T-cell infiltration seems to be a characteristic of the subset of vaccinated patients who underwent reoperation.

### Intradermal *vs* intratumoral and intradermal administration comparisons

As shown in Table 4

**Table 4:** Intradermal *vs* intratumoral and intradermal administration comparisons

**Case**	**DTH reaction to tumour lysate**	**ELISPOT assay**	**Radiological findings**
*Intradermal administration*			
1	−	ND	NC
7	ND	−	PD
8	+	+	NC
9	ND	−	PD
10	−	ND	PD
*Intratumoral and Intradermal administration*			
2	ND	ND	NC
3	+	−	MR
4	ND	ND	NC
5	−	ND	PD
6	+	+	MR

+=positive; −=negative; ND=not tested; MR=minor response; NC=no change; PD=progressive disease.

, five patients (Cases 1, 7, 8, 9, and 10) had intradermal administration, and five other patients (Cases 2, 3, 4, 5, and 6) had both intradermal and intratumoral administration. In the intradermal administration group, DTH reaction to the tumour lysate was positive in one of three tested cases, ELISPOT assay was positive in one of three tested cases, and there were two NCs and three PDs radiological findings after vaccination. In the intradermal plus intratumoral administration group, DTH reaction to the tumour lysate was positive in two of three tested cases, ELISPOT assay was positive in one of two tested cases, and there were two MRs, two NCs and one PD radiological findings after vaccination. A clinical response was observed in the intradermal plus intratumoral administration group.

## DISCUSSION

Dendritic-cell-based therapy is currently being evaluated in clinical trials for a variety of tumours. The initial results obtained from clinical trials of DC-based therapy for B-cell lymphoma (Hsu *et al*, 1996), melanoma (Nestle *et al*, 1998), prostate cancer (Tjoa *et al*, 1998), and renal cell carcinoma (Kugler *et al*, 2000) have recently been published, and the results appear to be encouraging. Antigen-specific immunity has been shown to be induced in the majority of patients during DC vaccination, and regression of metastases was observed in 30% of patients (Nestle *et al*, 1998). The remarkable ability of DCs to elicit immune responses and the availability of DC culture systems has allowed the use of DCs in cancer immunotherapy. Large numbers of functional DCs can be isolated from bone marrow precursor cells treated *in vitro* with cytokines such as GM-CSF, IL-4, and tumour necrosis factor (TNF)-*α*. Together, these findings demonstrate that autologous DCs from tumour-bearing hosts can be expanded *ex vivo*, pulsed with tumour antigens, and reintroduced to induce tumour-specific T cells. In murine models and in clinical trials, these cytokine-stimulated DCs have been successfully pulsed *ex vivo* with tumour antigens for use as antitumour vaccines against several types of malignancies.

In this Phase I/II trial, the patient's peripheral blood DCs were pulsed with the autologous tumour lysate of the glioma. The mean numbers of vaccinations were 3.7 times intradermally close to a cervical lymph node, and 3.2 times intratumorally via the Ommaya reservoir. There were two minor responses and four no-change cases, as evaluated by radiological findings. Dendritic cell vaccination elicited T-cell-mediated antitumour activity, as evaluated by the ELISPOT assay, after vaccination in two of five tested patients. These two patients who demonstrated CTL activity in the ELISPOT assay had MR or NC response, as evaluated by MRI. There was a significant increase in CD56-positive cells in PBL after vaccination, suggesting that DC therapy activated systemic NK cells. Intratumoral CD4+ and CD8+ T-cell infiltration was detected in two patients who underwent reoperation after vaccination. These patients who underwent reoperation demonstrated MR or NC response, as evaluated by MRI. Case 3 and Case 6 patients, who received intradermal and intratumoral injections, both had a positive DTH reaction and a minor clinical response. Intratumorally injected DCs may induce more efficient antitumour immunoresponses. This study demonstrated the safety, antitumour immunoresponses, and clinical response of autologous tumour lysate-pulsed DC therapy for patients with malignant glioma. Furthermore, in our *in vitro* study, DCs pulsed with a normal brain lysate failed to induce cytolytic T-cell activity against autologous glioma cells, suggesting the lack of an autoimmune response (data not shown). There were also no serious adverse effects, or clinical or radiological evidence of autoimmune reaction, in any of the patients in this study. Although there is some probability of shared antigens between tumour and normal CNS tissue, and possible contamination of normal tissue in the tumour lysate, we have not observed autoimmunity either *in vitro* or *in vivo*.

Intratumorally injected DCs would acquire and process tumour antigens *in situ*, migrate to regional lymphoid organs via lymphoid vessels, and initiate a significant tumour-specific immune response. It has been reported that DCs have antigen-capturing and processing as well as trafficking abilities only during their immature phase (Inaba *et al*, 1993). We generated immature phase DCs by GM-CSF and IL-4, without TNF-*α*. In all cases, the DC preparation was loaded with KLH protein, since KLH has been shown to serve as a strong surrogate antigen and an immunogenic marker for immunisation studies using DC-based vaccines (Hsu *et al*, 1996; Nestle *et al*, 1998).

There have been several reports (Liau *et al*, 1999; Heimberger *et al*, 2000; Akasaki *et al*, 2001; Aoki *et al*, 2001; Ni *et al*, 2001; Yamanaka *et al*, 2001, 2002; Insug *et al*, 2002; Witham *et al*, 2002) on murine glioma models for DC-based therapy. These animal studies of DC therapy for glioma differ from each other. These differences are the source of the DCs, type of antigen, administration route, dose, and number of vaccinations. The antigens used varied, and included tumour lysate admixed with liposomes, whole-tumour mRNA, whole-tumour peptides, virus vector-mediated tumour cDNA, glioma apoptotic bodies, and fusions of DCs and tumour cells. Most investigators used bone marrow DCs as the source of DCs, except for one cloned DC line. The administration route was intraperitoneal, except for one case of subcutaneous administration. They reported that DC therapy led to prolonged survival in rodents with established tumours, and also induced specific cytotoxic activity. No severe autoimmune responses occurred in any of the studies using animal models. Therefore, DC-based systems are expected to serve as powerful tools for treating patients with malignant glioma.

There have been two reports (Kikuchi *et al*, 2001; Yu *et al*, 2001) of DC-based clinical trials of vaccination of glioma patients. The source of the DCs in these cases were PBMCs cultured with GM-CSF, IL-4, and TNF-*α*. Dendritic cells were stimulated with tumour antigens using peptides eluted from autotumour cells, or fusions of DCs and autotumour cells. The administration route was intradermal injection close to a cervical lymph node such as the deltoid or cervical region. Patients were monitored for toxicity, immunological response, tumour size as determined by MRI or CT, and survival.

Yu *et al* (2001) reported a phase I trial of peripheral blood DCs pulsed with peptides eluted from the surface of autologous glioma cells. Five women and four men were enrolled in this study. Two patients had anaplastic astrocytoma and seven had glioblastoma. Prior to DC treatment, patients had undergone surgical resection of the tumours followed by irradiation therapy. A total of 1 million peptide-pulsed DCs were injected intradermally in the deltoid region three times biweekly. Dendritic-cell vaccination elicited systemic cytotoxicity in four of seven patients, and intratumoral cytotoxic and memory T-cell infiltration was detected in two of four patients who underwent reoperation after vaccination. No significant adverse effects were seen. They also reported increased survival: the median survival times for the study and control groups were 455 and 257 days, respectively.

Kikuchi *et al* (2001) reported immunotherapy with fusion of DCs and glioma cells. Eight patients with malignant glioma participated in this study. Dendritic cells were generated from peripheral blood, and cultured autologous glioma cells were established from surgical specimens in each case. Fusion cells produced using dendritic and glioma cells were prepared with polyethylene glycol with an average fusion efficiency of 21.9%. Patients received the fusion cells every 3 weeks intradermally close to a cervical lymph node. The number of fusion cells injected ranged from 2.4 × 10^6^ to 8.7 × 10^6^ cells per vaccination. The percentage of CD16+/CD56+ cells and the production of IFN-*γ* in peripheral blood lymphocytes increased after immunisation. Clinically, there were no serious adverse effects and two partial responses. Magnetic resonance imaging of two cases showed that, in the first case, the size of the primary tumour was decreased although there was progression of the secondary lesion after immunisation. In the second case, although the size of the tumour itself had not changed, a reduction of the brain oedema was observed.

Dendritic-cell vaccination of patients with glioma appears to be safe and not associated with autoimmunity. Toxicities included mild headache in one patient in our study. The only adverse effects reported were mild fever and swelling of an injected lymph node (Nestle *et al*, 1998), and no other significant adverse effects have been reported in DC therapy clinical trials, or in animal models of DC therapy. Due to the limited populations studied thus far, further evaluation of DC immunotherapy is necessary in order to determine the optimum dose of DCs, the appropriate route of vaccinations, the best source of tumour antigens, and methods of antigen loading.

Phase II studies of DC-based glioma therapy are underway to confirm its effects on survival in patients with malignant glioma. The DC-based immunotherapy strategy appears promising as an approach for inducing antitumour immune responses and a clinical response in patients with glioma. The efficacy of such protocols should be determined in randomized, controlled clinical trials. The development of methods for manipulating the vaccination of DCs will enhance the clinical usefulness of DC-based biotherapy for malignant glioma.
